# Integrating Artificial Intelligence into Ventilation on Demand: Current Practice and Future Promises

**DOI:** 10.3390/s26031042

**Published:** 2026-02-05

**Authors:** Chengetai Reality Chinyadza, Nathalie Risso, Angel Aramayo, Moe Momayez

**Affiliations:** School of Mining Engineering and Mineral Resources, University of Arizona, Tucson, AZ 85721, USA; cchinyadza@arizona.edu (C.R.C.); aaramayo@arizona.edu (A.A.); mmomayez@arizona.edu (M.M.)

**Keywords:** ventilation on demand, artificial intelligence, cyber–physical systems

## Abstract

The increasing depth and complexity of underground metal mining has raised ventilation energy demands and safety risks, driving the need for intelligent and more adaptive ventilation systems. Ventilation on Demand (VOD) systems dynamically adjust airflow using real-time operational and environmental data to improve energy efficiency while maintaining safety. Although VOD has been applied for over a decade, deeper and more extreme mining environments associated with critical minerals extraction introduce new challenges and opportunities. VOD systems rely on the tight integration of hardware, sensing, optimization-based control, and flexible infrastructure as mining operations evolve. The application of Artificial Intelligence (AI) introduces significant opportunities to further enhance and adapt VOD systems to these emerging challenges. This work presents a comprehensive review of the state of the art in AI integration within VOD technologies, covering sensing and prediction models, control strategies, and optimization frameworks aimed at improving energy efficiency, safety, and overall system performance. Findings show an increasing use of hybrid deep learning architectures, such as CNN-LSTM and Bi-LSTM, for forecasting, as well as AI-enabled optimization methods for sensor and actuator placement. Key research gaps include a reliance on narrow AI models, limited long-term predictive capabilities for maintenance and strategic planning, and a predominance of simulation-based validation over real-world field deployment. Future research directions include the integration of generative and generalized AI approaches, along with human–cyber–physical system (Human-CPS) designs, to enhance robustness and reliability under the uncertain and dynamic conditions characteristic of deep underground mining environments.

## 1. Introduction

**Figure 1:**
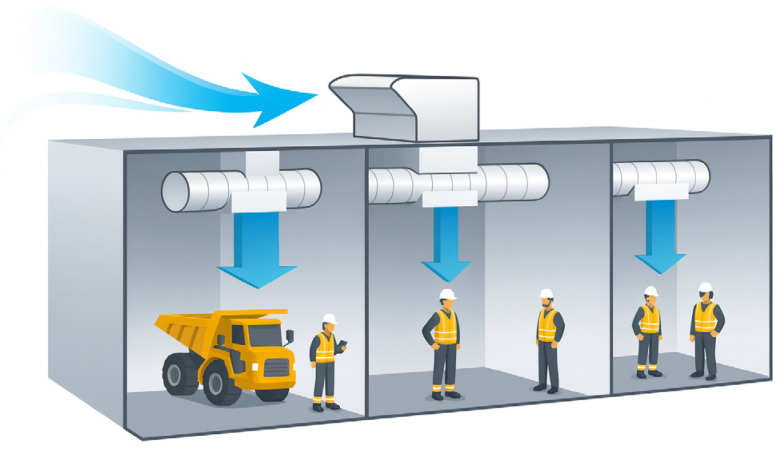
Conceptual representation of Ventilation on Demand.

Increased population, energy use, and technology democratization are driving an unseen demand for critical minerals [1]. This demand, along with declining ore grades, is shifting the mining industry from surface to underground methods, and it is motivating the development of deeper mines more efficiently and safely. Mineral deposits occur worldwide under diverse environmental conditions, often exposing underground operations to extreme temperatures that require effective ventilation and temperature control. Underground mine ventilation is essential for maintaining a continuous supply of fresh air to dilute and remove contaminants, such as diesel particulate matter (DPM), carbon monoxide (CO), nitrogen oxides (NOx), methane, and dust, while ensuring adequate visibility and thermal comfort, both of which are vital for safe and productive operations [2]. As mining activities extend to greater depths, energy consumption and associated operational expenditures (OPEX) are rising considerably. Currently, ventilation systems can account for up to 50% of total electrical power usage ref. [3] and approximately 15% of overall operating costs in a mining operation ref. [4], thus reinforcing the need for optimized ventilation. To address these challenges, Ventilation on Demand (VOD) has emerged as an alternative for improving energy efficiency and costs. VOD dynamically regulates airflow based on real-time operational conditions, such as, for instance, personnel location, equipment activity, gas concentrations, and temperature variations. This enables control of both local and mine-wide ventilation, ensuring that airflow is delivered only where and when it is needed ref. [5] (Figure 1). By aligning ventilation delivery with real-time operational needs, VOD optimizes energy use while keeping worker comfort and safety and regulatory compliance [6]. This strategy has reported energy savings of up to 43% [7]. VOD can, then, be extremely valuable in operations where ventilation demand is highly variable, energy is a major operating cost, and energy and performance tradeoffs can be improved by targeted airflow control [5,8].

VOD adoption has expanded extensively worldwide, although the literature does not consistently report a definitive count of fully deployed systems. Major technology providers, including ABB, Epiroc, and Howden, have introduced advanced VOD solutions in leading mining operations, such as Garpenberg (Sweden), Borden (Canada), Chuquicamata (Chile), and Onaping Depth (Canada). These implementations, although in very different regions and climates, manage to leverage real-time monitoring, automated control, and data-driven optimization to improve both energy efficiency and underground safety [8]. In practice, effective VOD systems rely on dynamically adjusting airflow in response to environmental inputs, such as real-time gas and dust concentrations; current airflow; and temperature, humidity, pressure, and operational factors, including personnel location, equipment status, and production activity [9,10]. System outputs typically include optimized fan speeds, damper adjustments, real-time alerts, and reports on energy savings and emission reductions depending on the operation needs [11]. VOD implementation, then, can vary considerably depending on the mine’s type, as well as the age, depth, and extraction method. VOD adoption pathways and challenges differ considerably between new (greenfield) and existing (brownfield) projects. Greenfield mines can integrate sensing, control, and digital infrastructure around VOD during design, whereas brownfield operations often depend on legacy static ventilation systems with limited data connectivity, constraining data-driven control optimization [12]. Mining methods also strongly influence VOD effectiveness. Intermittent operations, such as room-and-pillar mining, exhibit irregular activity patterns and localized work zones where personnel and equipment move in and out [10]. These conditions enable stratified airflow management, allowing VOD to modulate ventilation based on occupancy or equipment activity, thus achieving meaningful energy savings [7]. In contrast, continuous methods, such as sublevel stoping, involve sequential and interconnected processes requiring consistent ventilation, which limits the feasibility of demand-based control. In deeper and more complex mines, VOD implementation presents additional challenges. As depth increases, the geothermal gradient raises ambient temperatures and heat loads, intensifying cooling requirements [13,14]. Similar requirements apply for operations located in extreme climate conditions, such as Resolution Copper (USA), where controlled cooling is needed [15], or operations transitioning from open pit to underground, such as Chuquicamata (Chile). Furthermore, the integration of diesel and electric equipment along with blasting by-products also contributes to intense heat and contaminant load, as well as an increased requirement in prediction and timely ventilation actuation capabilities. VOD systems address this by adapting airflow based on depth, workforce distribution, and environmental conditions [16,17]. However, achieving this requires accurate, real-time prediction of airflow needs and continuous environment monitoring under these new conditions. Moreover, complex mine geometries with branching tunnels, evolving headings, and variable resistance pathways necessitate optimizing strategies for proper sensor placement and adaptable control strategies to maintain ventilation efficiency [18,19]. In addition, VOD requirements become even more challenging when it comes to integrating them into mine planning. In Dynamic Mine Planning (DMP), for instance, production schedules adapt to changes in the operating environment, such as market fluctuations, equipment failures, or evolving geological conditions. Integrating energy efficiency into predictive decision making requires timely updates and accurate forecasting, which can be challenging in large, coupled, and distributed systems like those used for VOD deployment. These unique challenges add strong requirements for VOD systems. Thus, infrastructure, real-time data processing, and predictive capabilities need to be adapted and improved ref. [20], in a case-specific manner, for VOD to be deployed at a larger scale and to comply with new industry requirements.

In recent years, and sustained by the digital transformation in the mining industry, VOD systems have taken increasing advantage of Artificial Intelligence (AI) to enhance their sensing, forecasting, and control decision making [21]. AI, in general, enables systems to perform tasks associated with human intelligence, such as learning, reasoning, problem solving, decision making, creativity, and autonomy refs. [22,23] which are tasks that are essential in several VOD components. AI branches include machine learning (ML), deep learning (DL), natural language processing (NLP), computer vision (CV), expert systems (EX), knowledge representation and reasoning (KRR), and robotics, all of which offer many valuable perception, prediction, and reasoning capabilities that can be used to overcome VOD challenges. In addition, modern AI also includes some more recent notions: Artificial Narrow Intelligence (ANI), Generative AI (GenAI), and Artificial General Intelligence (AGI) [24]. ANI focuses on specialized, task-specific applications; GenAI produces human-like outputs, such as text, images, and code; and AGI seeks human-level reasoning, adaptability, and self-learning. Across all these forms, AI offers promising opportunities, in terms of safety, efficiency, and predictability, to enhance VOD systems [2,19].

### Contribution

Although many works in the past have been devoted to report advancements in the automation and improvement in VOD systems, both from an industry and academic perspective, none have approached the topic from the perspective of assessing the existing contributions to VOD that rely on AI methods. In particular, this work presents a subsystem-based synthesis of AI-enabled Ventilation on Demand systems (VOD) that are framed explicitly as cyber–physical systems (CPS). This explicit consideration is intended to highlight the interdisciplinary nature of VOD system design, which relies on mining engineering principles, hardware–software integration, and the proper use of data-driven methods to sustain effective and timely decision making, particularly when it involves the use of AI methods. Thus, the intended audience of this work includes AI/CPS researchers and developers, seeking to understand where and how AI can benefit VOD systems to motivate further research and development, as well as how to benefit mining and ventilation practitioners seeking a structured, evidence-based view of current AI capabilities, limitations, and deployment needs in underground ventilation systems. To accomplish this, we organize the evidence across intelligent sensing/forecasting, actuation, and control/decision making; map reported approaches to the ANI–GenAI–AGI spectrum to distinguish mature capabilities from emerging directions; and assess surface system-level barriers to scale-up, including short prediction horizons, limited field validation, and underdeveloped Human-CPS design (trust, override logic, and operational procedures). As most published and commercial implementations, to date, emphasize narrow ANI-driven functions rather than end-to-end VOD intelligence, this review consolidates what is known and what is missing across the full VOD workflow—from sensing and prediction to control and optimization—with the goal of improving energy efficiency, safety, and system performance. Specifically, we: (1) quantify where AI is being deployed across VOD tasks and subsystems; (2) synthesize the dominant technical and operational challenges reported in the literature; and (3) outline a focused research agenda for next-generation AI-driven VOD suitable for increasingly deep, complex underground environments.

## 2. Background

This section provides a brief functional description of VOD and its subsystems. The aim is to present a framework that allows for a functional and systemic description of existing AI tools used to solve VOD design and implementation challenges.

### 2.1. Ventilation on Demand

**Figure 2:**
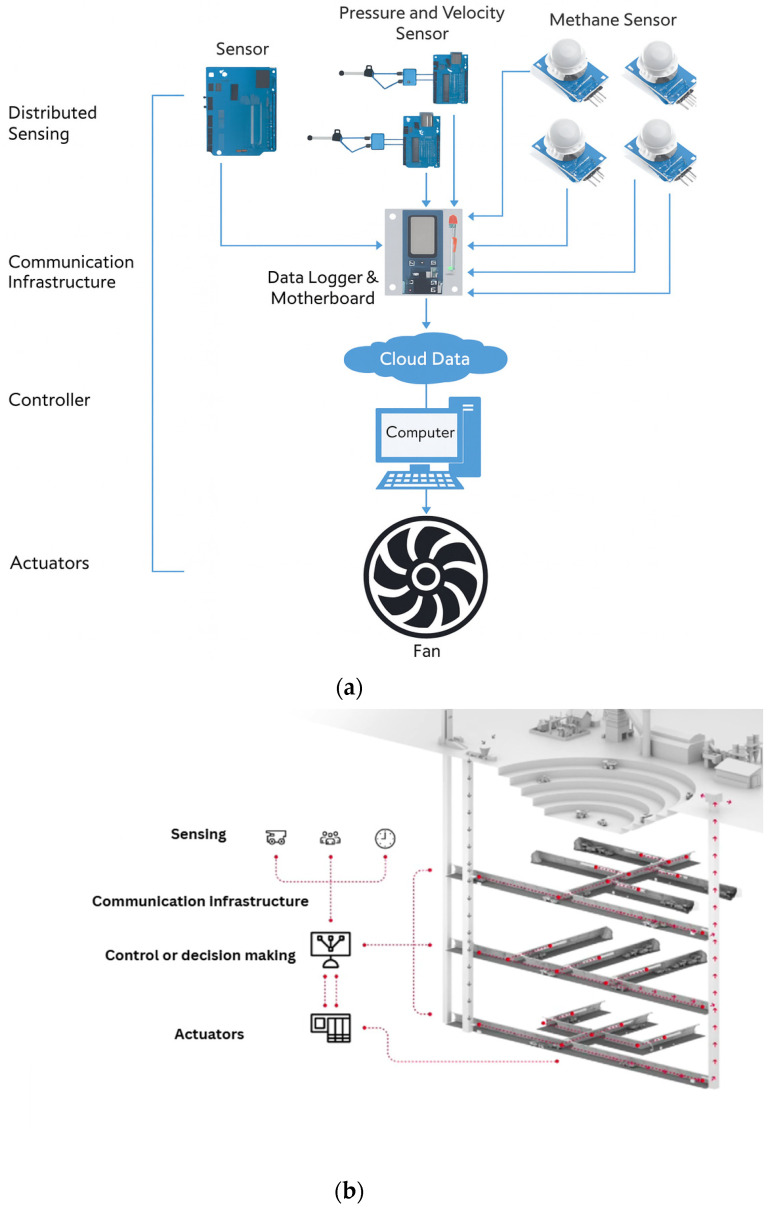
(**a**) Example application of VOD as a cyber–physical system (adapted from [26]). (**b**) Example of an industrial VOD platform architecture (adapted from the ABB Ability™ Ventilation Optimizer, ABB, Zurich, Switzerland (Ventilation on Demand for underground mines) official ABB website).

VOD systems are classified as cyber–physical systems because their effective design and operation depend on the tight integration of computational intelligence with physical ventilation processes to enable coordinated, intelligent control [25]. VOD systems can be described in terms of four functional subsystems: sensing; actuation; control and decision making; and communications. An example of how each CPS component is represented in VOD is provided in Figure 2. Figure 2a provides an example of a development prototype for VOD design, while Figure 2b presents an example of a packaged solution for the VOD currently available from an industry provider. Note that, both in the development and the packaged solution, the components and integration of CPS systems are highlighted.

While these example CPS systems do not explicitly portrait human-in-the-loop interactions, VOD control remains human-supervised in most operations. This introduces additional operational challenges, including those related to system acceptance, the establishment of standard operating procedures, and the definition of hierarchical override structures for supervised control (operator, supervisor, and ventilation engineer) that incorporate appropriate guardrails and audit trails. We next provide a brief overview of each of the VOD subsystems.

#### 2.1.1. Sensing

Consider all the hardware and software used for the collection and processing of real-time operational and environmental data. Typically, VOD systems track microclimate conditions, personnel numbers, vehicle locations, air quality parameters (like oxygen, methane, carbon monoxide, and nitrogen oxides concentrations, as well as particulate matter, temperature, and humidity and barometric pressure), and actuator states. This includes detecting regulator positions, refs. [4,25,26,27] and real-time personal and equipment tracking [28]. These tracking devices oftentimes use beacons (tags) affixed to equipment, as well as those fixed to locations or carried by personnel [29,30]. In addition to hardware, sensing also considers perception capabilities deployed as smart and soft sensors with data-driven capabilities that perform forecasting of relevant variables, for example, wind-speed sensor signals can be denoised using CEEMDAN–wavelet thresholding to improve data quality prior to analysis and prediction [31]. Sensing infrastructure, both in terms of components and their locations, plays a crucial role in the quality of monitoring, forecasting, and control for VOD systems, thus representing an important design element [32].

#### 2.1.2. Actuators

**Figure 3:**
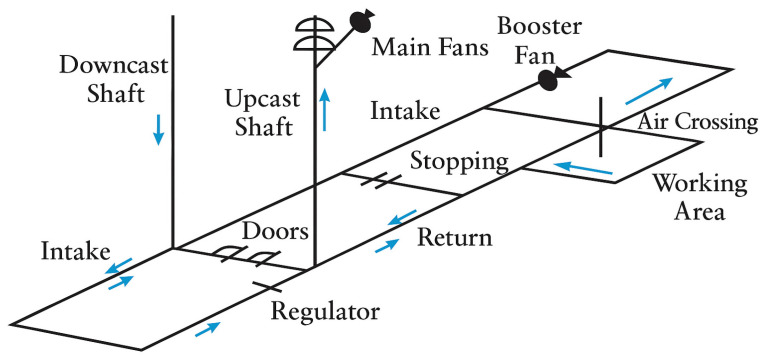
Underground mine ventilation system components (adapted from [33]).

Actuators translate digital signals into mechanical actions to adjust airflow according to requirements. They implement the control and decision-making subsystem commands into real-life actions over the physical infrastructure (dampers, fans, and regulatory devices, see Figure 3). Different VOD actuators include the following.

- Primary ventilation fans: These serve as the backbone of mine ventilation systems [33]. They are the main providers of bulk airflow, pushing fresh air into the mine and pulling contaminated air out. They use 25-to-50% of total system energy [2].
- Variable Speed Drive (VSD) fans: These dynamically adjust their rotational speed to align with real-time ventilation demands. Their operation is governed by the fan affinity laws, which establish that power consumption varies proportionally to the cube of the fan’s rotational speed [33,34]. This highlights the capability of VSD technology to optimize energy while maintaining safety [35,36].
- Automated Dampers: These are responsible for airflow routing and isolation in VOD systems. They allow dynamic reconfiguration of ventilation networks to curb changes in operational conditions. Automated dampers and ventilation doors vary from butterfly dampers (used to provide variable flow control with low pressure drop), Louver damper (used to isolate sections of a mine), ventilation doors (used to isolate for maintenance and emergency conditions), and automatic regulators (used to maintain pressure differences across critical boundaries) [37,38].

#### 2.1.3. Control and Decision Making

This subsystem must provide answers to practical questions about *where* ventilation is needed, *how much* air is to be supplied, and *when* to change it. It relies on inputs from sensing and forecasting to determine commands [39]. For implementation, determining network flow and resistance flow models (nodal pressure methods) remain foundational in airflow dynamics, ref. [40] as well as pressure drop and regulator settings, in most underground mines [41]. These models are typically used for balancing airflow and simulating the impact of adjustments. As mine digitalization increases, these methods are also complemented by the use of operational data and can integrate optimization approaches, such as energy minimization under hard safety constraints [11,42]. Examples of this include fan speed control based on demand and time-of-use tariffs to guide energy-aware scheduling [30].

#### 2.1.4. Communication

Communication and networking infrastructure is essential in VOD systems as it enables data transmission from distributed sensors to control and alarm systems, thus forming the backbone of real-time control [43]. The VOD Communication system is briefly described in the CPS as the enabling infrastructure of the VOD system; however, AI methods whose primary objective is communication or network optimizations are outside the scope of the study. The interested reader is referred to ref. [44] for broader discussion on the selection and design of underground mine communication infrastructures and to the comprehensive review in ref. [45] for recent advances related to IoT integration into VOD.

Although out of scope, ref. [46] establishes that VOD communication networks must reliably support the coordinated control of ventilation devices and robust system operation across distributed nodes. Ref. [45] highlights the necessity of these networks for assessing environmental variables through real-time sensor data. According to refs. [44,47], critical design considerations include cybersecurity, network topology, and high availability. Ref. [45] further emphasizes the importance of communication protocols, low latency, and sufficient bandwidth. To manage communication failures, ref. [46] discusses utilizing programmable units to buffer data locally for later transmission. Finally, refs. [45,47] describe the use of automated gateways and alarms, such as buzzers or messages, to notify personnel of hazardous conditions and ensure safe operation.

## 3. Review Methodology and Materials

This review examines the impacts of AI across sensing, actuation, and control subsystems of VOD systems, which are framed as a CPS. Communication and networking systems are included as enabling infrastructure that links the subsystems; however, we excluded from our review AI integration over communication infrastructure, as a recent review on this topic can be found in [45]. Results from this review complement our findings, which are described in the final section.

The research was conducted via the following four steps:

1. Defining review scope and research questions.
2. Identifying and acquiring records.
3. Screening of the records.
4. Reviewing literature.

### 3.1. Scope and Research Questions

The scope of this review is to provide an understanding of where underground mine ventilation stands with respect to AI-enabled VOD technologies when framed as a CPS. The research questions considered to bound the review and guide reference search are listed next (Table 1).

### 3.2. Search Strategy

Following the PRISMA guideline ref. [48], titles and abstracts were screened independently by two reviewers against the inclusion and exclusion criteria listed in Table 2. Full-text articles of potentially eligible records were subsequently assessed independently by the same reviewers, with disagreements resolved through discussion. No automated screening tools were used. With these criteria, a structured search strategy was implemented across multiple databases using combinations of Boolean operators and targeted key phrases. For example, a search string (Scopus; TITLE-ABS-KEY): (“ventilation on demand” OR “VOD” OR “mine ventilation control”) AND (“artificial intelligence” OR “machine learning” OR “deep learning” OR “reinforcement learning” OR “neuro-fuzzy”) was used. In addition to database search, identified studies found through citation chaining were also included, leading to approximately 65% from database search results and 35% from backward/forward citation chaining.

### 3.3. Data Extraction, Outcomes, and Risk of Bias Assessment

Data was extracted using a predefined extraction framework. Extracted variables included mine type, VOD subsystem, AI methodology, data sources, validation level, and implementation scale, where reported. Outcomes of interest included ventilation performance, energy efficiency, AI model performance metrics, and operational applicability. All results compatible with each outcome domain were collected as reported, and missing or unclear information was recorded as not reported and not inferred. Risk of bias was assessed independently by three reviewers using a modified Cochrane framework for experimental studies and a customized checklist for AI- and simulation-based studies, focusing on data quality, validation rigor, transparency, and applicability to real mining environments.

### 3.4. Overview of the Literature Retrieved

**Figure 4:**
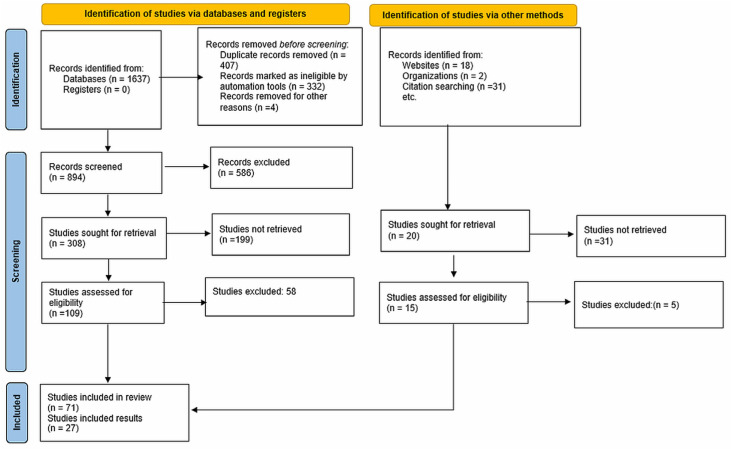
PRISMA flow diagram used for systematic review.

Using the above strategies, this work synthesizes 71 publications spanning relevant to the topic of study. Figure 4 provides an overview of records included and excluded and their identification methods. The records retrieved and considered for the study fall in general across the following categories.

- Mining-specific journals: 15 records, including *Mining, Metallurgy & Exploration*; *Mining Report*; *Journal of the Southern African Institute of Mining and Metallurgy*; *International Journal of Mining Science and Technology*; *Mining Technology*; *Mining*; *American Journal of Mining Engineering*; *Academic Journal of Science and Technology*; *Journal of Mining Institute*; and *Archives of Mining Sciences*.
- General engineering journals: 24 records, including *IEEE Access*; *Processes*; *Procedia CIRP*; *Energies*; *Applied Energy*; *International Journal of Modern Research in Engineering and Technology*; *Measurement*; *Sensors*; *Mathematics*; *Applied Sciences*; *Journal of The Institution of Engineers (India) Series D*; *Sensors*; *Scientific Reports*; *International Journal of Low-Carbon Technology*; *IEEE Sensors Journal*; *Procedia Computer Science*; *Journal of Wind Engineering and Industrial Aerodynamics*; *Computers and Electrical Engineering*; and *Machines*.
- Journals with focus on AI tools: 5 records, including *IEEE Access (AGI survey)*; *Engineering Applications of Artificial Intelligence*; *Expert Systems with Applications*; and *Frontiers in Artificial Intelligence*.
- Health and safety-focused journals: 5 records, including *Journal of Loss Prevention in the Process Industries*; *Process Safety and Environmental Protection*; and *International Journal for Housing Science and Its Applications*.

This preliminary assessment shows an approach to VOD in mining from different disciplines, also spanning different degrees of depth and detail in the description of AI integration. This source distribution reinforces the notion of analyzing VOD systems from a multidisciplinary perspective, as is common in other cyber–physical system applications.

### 3.5. AI Use Disclosure

In addition to the methods described above, the authors of this paper took advantage of Generative AI tools, such as ChatGPT 5.0 and Notebook 2.0, to aid in the visualizations included in this paper (Figure 1, Figure 2 and Figure 3). Additionally, ChatGPT was used for minor grammatical and stylistic editing of the manuscript. No AI tools were used for data generation, analysis, or interpretation.

## 4. Results

Findings from publication records considered in this study are presented in this section. Results are organized around the research questions defined in the previous section and described in terms of VOD subsystems, when appropriate.

**Figure 5:**
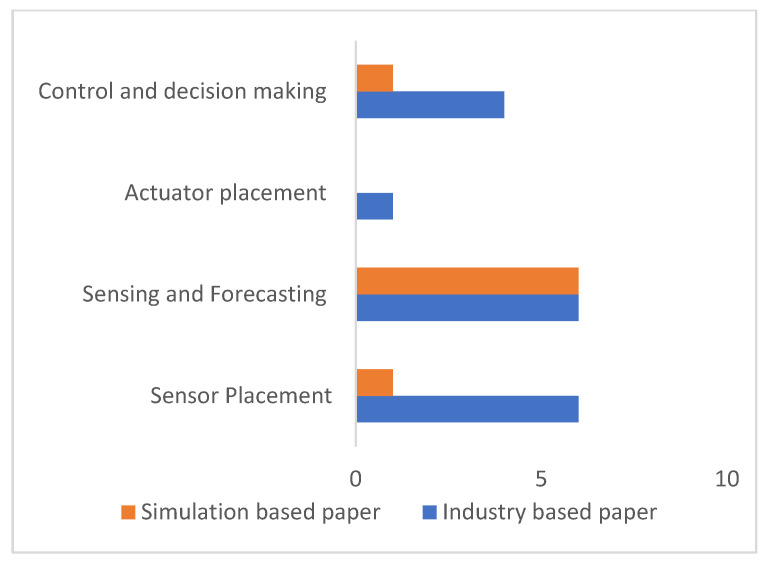
Distribution of the studies in the results section across the VOD subsystem components.

Figure 5 provides a high-level overview of the evidence base synthesized in the Results section. The bar chart groups the reviewed studies according to the primary VOD subsystem or task they address, their Sensor placement, Sensing and forecasting, actuator placement strategies, and Control/decision making, and it also distinguishes the validation context of each study. Specifically, Industry-based papers correspond to work validated using real mine data, operational deployments, or field-relevant implementations, whereas Simulation-based papers reflect studies evaluated primarily through modeling, numerical simulation, or laboratory-scale testing. We have kept both types of articles as they are representative of industry-deployed solutions and advancements in terms of research and development at lower TRLs, respectively. This summary makes the balance of evidence transparent and helps frame the interpretation of the subsequent tables: it highlights where the results are supported by field validation versus where the literature remains dominated by simulation-driven research, providing context for the gaps and deployment challenges discussed later.

### 4.1. Operational and Analytical Domains of AI Integration in VOD

In addressing Research Question Q1, the results show that the AI tools were integrated into all the subsystems and tasks of VOD within the scope of this paper (although not to the same extent). In many of the reported cases, AI implementation is integrated into one or more subsystems, which highlights the CPS framework associated with VOD systems design and deployment. A crucial aspect is the prediction of requirements and forecasting in mine ventilation systems, which are challenging to resolve using conventional deterministic methods [49]. As shown in Figure 5, the sensing and forecasting subsystem is dominated by simulation-based studies relative to the other VOD components, indicating that field validation remains comparatively limited in this area.

### 4.2. AI Techniques and Models Applied in VOD Systems

This section describes results associated with Research Questions Q2 and Q3. Since AI has been applied across all VOD subsystems, often in more than one of them, we have organized the results identifying AI tools in terms of the main subsystem, which is targeted on the research literature. In this manner, results are presented in terms of: sensor placement strategies (Table 3); sensing and forecasting (Table 4); actuator placement strategies (Table 5); and controllers and decision making (Table 6). In synthetizing content and identifying the AI methods used, we have provided for each application case in terms of the available information associated with the variables used as inputs and outputs, the dataset information (when provided), the specific AI models and methods, and the performance metrics in each study.

#### 4.2.1. Sensor Placement Strategies

The principles for placing sensors in intelligent mine ventilation networks center on achieving comprehensive network visibility while maintaining cost-effectiveness and resource minimization [51,64]. A primary objective is determining the minimum number and location of sensors necessary for well-posed airflow reconstruction, which involves accurately inferring the airflow throughout the entire system from limited measurements [51]. Sensor placement is highly strategic, focusing on sensitive branches ref. [25]; mandatory monitoring locations, like main return tunnels ref. [51]; and points that enable intelligent adjustment of main and auxiliary fan speeds for VOD systems [28,65]. Ultimately, this strategic data collection supports the mathematical models used for analyzing the network structure and solving the ventilation network to select optimal operating parameters [28,51].

Recent studies have been identifying several strategies for sensor placement optimization in mine ventilation networks (Table 3). The surveyed works employ narrow AI tools in general to solve heuristic optimization and determine optimal number of sensors and their placement. Common inputs for optimization include ventilation network layout (VNL), ventilation network graph (VNG), number of sensors (NS), and airflow volume and direction, as well as airflow time. Gas concentration and pressure losses are also considered in [50]. Performance is assessed in general in terms of the minimum number of sensors, flow reconstruction, reliability, and response time, among others. Main methods found in the literature include genetic algorithms (GAs); ant colony optimization (ACO) and variants; evolutionary methods and local search algorithms; and decision trees (DTs). In particular, ref. [50] proposes the Efficient Placement of Air Quality Sensors (EPAQS) algorithm to locate air quality sensors to maximize statistical coverage of ventilation zones with no redundancy. The paper addresses the challenge of intelligently regulating ventilation networks, particularly by proposing the concept of frequency sensitivity analysis to identify the most responsive air branches. It is validated via simulation using a ventilation model from an underground coal mine in Utah. This represents research-based evidence aimed at establishing best-practice frameworks. Ref. [52] employs decision rule-based placement based on DT, with sensors deployed per operating needs, such as shunt disturbance avoidance and complete coverage of key nodes, along with historical data, and classification conditions to determine sensor location. The DT algorithm here presents limitations stemming from data quality/quantity, constraints inherent in the algorithm itself, and application challenges. These include, but are not limited to, small sample sizes to train models, overfitting, and inconsistencies in data type resulting in wrong optimal solution. The research was validated through field engineering applications, providing industry-based evidence. In ref. [51], an efficient graphical algorithm for optimizing the placement of wind speed sensors and reconstructing air volume is proposed. A key innovation is the independent cut set, which leverages the mine’s graph structure to determine the minimum number and optimal placement of sensors for well-posed flow reconstruction. A coal-mine case study shows that it requires sensors in fewer than 30% of tunnels, while still accurately estimating airflow everywhere, thus reducing installation and maintenance costs. This research was validated via simulation using real mine datasets, providing research-based evidence. Combined, these methods emphasize balancing cost, coverage, and observability based on algorithmic optimization to real-world guidelines for engineering.

#### 4.2.2. Sensing and Forecasting

In VOD systems, the inputs for decision making are critical for effective performance. Mine ventilation networks are complex, nonlinear, and operate under significant uncertainty, making them difficult to model with traditional deterministic or rule-based models. In contrast to rule-based methodologies, AI/ML approaches learn from data, detect patterns, and adapt to changing conditions without explicitly programmed rules. This makes them well suited for forecasting nonlinear ventilation behaviors, handling uncertainty in underground environments, and responding to the dynamic conditions of mining operations.

Sensing and forecasting has taken advantage of several machine learning and deep learning implementations. Problems are most often formulated as a regression with common forecasting outputs, including gas concentration and airflow over a future window (typically 30–60 min). In particular, Convolutional Neural Network (CNN) and Long Short-Term Memory (LSTM) networks have been used in hybrid architectures to predict local and global airflow variations. Ref. [36] proposed a 1-Dimensional Convolutional Neural Network-Long Short-Term Memory) architecture that is deep learning model designed for the precise prediction of airflow parameters by providing the spatial framework (EPAQS) required to ensure the sensors feeding the engine are optimally positioned. The paper is simulation-based but includes validation through a laboratory-based prototype setup. Ref. [66] proposed a dual-layered ventilation framework, in which a deep learning-informed Koopman approach forecasts mine airflow dynamics by linearizing nonlinear behavior for improved gas concentration prediction under changing wind conditions, while a Fuzzy-PID controller uses fuzzy logic to adapt PID control to uncertainty and complex underground dynamics for robust ventilation regulation. Ref. [66] proposed a dual-layered ventilation framework, in which a deep learning-informed Koopman approach forecasts mine airflow dynamics by linearizing nonlinear behavior for improved gas concentration prediction under changing wind conditions, while a Fuzzy-PID controller uses fuzzy logic to adapt PID control to uncertainty and complex underground dynamics for robust ventilation regulation.

Bi-directional LSTMs (Bi-LSTMs) have been used to improve predictions in gas concentrations [67]. Ref. [57] proposed a dynamic gas-concentration prediction framework that uses correlation-based indicator selection with spatiotemporal feature extraction and a hybrid Bi-LSTM/LSTM architecture to forecast hazardous buildups near a mechanized longwall face. They validated the model using multi-sensor monitoring data from an operating underground longwall (36 days), demonstrating improved prediction performance over baseline methods.

In ref. [58], the authors used combined t-distributed Stochastic Neighbor Embedding (t-SNE) for dimensionality reduction along with a variational auto-encoder (VAE) for feature extraction and de-noising. This architecture is used to forecast concentrations of multiple gases in sealed-off areas. In addition to the hybrid models listed above [66], the deep learning Koopman to model concentration dynamics under different airspeeds has also been proposed, informing digital-twin studies and controller design, and it has been validated on a CFD-guided laboratory platform. Lastly, ref. [68], proposes a hybrid network that fuses the spatial information extracted by the 1D-CNN and feeds a LSTM to extract the temporal dependencies of the gases concentration to perform real time forecasting corrections using reinforcement learning. The overall forecasting performances reported across different works have provided generally good performances.

#### 4.2.3. Actuator Placement and Coordination Strategies

The optimal placement of fans and dampers directly impacts energy consumption, air quality, and overall system performance [69]. The complexity of mine ventilation networks has driven the development of sophisticated optimization approaches for actuators placement strategies (Table 5). Although the literature within the scope of this work that uses AI and ML methods is limited, the main applications target optimization in terms of energy use and coverage efficiency. Surveyed works use evolutionary computation methods, ref. [27], and traditional optimization approaches [60]. Fuzzy-based models are also employed for decision support, as described in [61].

#### 4.2.4. Control and Decision Making

Recent advances in Ventilation on Demand (VOD) systems for underground mines demonstrate a progression from optimization-based methods to adaptive neuro-fuzzy architectures for intelligent airflow control. The main control goals remain focused on airflow distribution refs. [19,62], maximizing energy efficiency refs. [26,63], and optimizing fan selection [61]. Ventilation network characteristics, air quality, gas concentrations, fan power, and environmental variables are used as control inputs to determine predictions and manipulated variables. Input information is determined from time series data in general and validated with different scenarios, with some studies including simulation and laboratory-based studies refs. [26,63] and others including validation from real life mine environments refs [19,61] or tunneling areas [62].

Control and optimization approaches include Multi-Objective Beluga Whale Optimization (MOBWO) for underground mine ventilation networks [19]. Here, the controller uses network-topology parameters, including the independent-circuit matrix pᵢⱼ, branch resistances Rⱼ, volumetric airflows qⱼ, and operational constraints on fan performance (Hᶠ, η) as part of the inputs to search for Pareto-efficient solutions that balance energy minimization with ventilation requirements. Reported fan-power reductions are between 10.3% and 21.1% relative to baseline operations. This framework exemplifies how metaheuristic optimization can systematically address network-scale energy management in mine-ventilation systems while respecting complex physical and safety constraints inherent to underground environments. Adaptive Neuro-Fuzzy Inference Systems (ANFIS), defined here as data-driven fuzzy controllers with trainable neural parameters, provide an alternative for VOD control that combines learning with interpretable rule bases tailored to underground-mine conditions [70,71]. The study in ref. [23] uses an ANFIS framework to develop an optimal fan prediction model and a model to estimate gas dilution time. Models are adjusted using real-time data to improve performance. The findings point at high potential for energy reduction (up to 43% under laboratory scale conditions tested). In ref. [23], Sugeno-type ANFIS models integrated with cloud infrastructure use real-time inputs (air pressure P, velocity v, methane concentration M, fan power FP, and dilution time t) to succesfully determine optimal fan-power setpoints and dilution-time forecasts with coefficients of determination R^2^ = 0.84 and 0.96, respectively. A hybrid deep learning framework combining Advanced Multi-head Cross Attention-based BiLSTM Network (AMCABN) and Multi-strategy Enhanced Mantis Search Algorithm (MEMSA) is proposed in [63] to optimize energy efficiency in a multifan underground mine. Here, real-time sensor data is used to predict ideal fan power requirements and to estimate the time needed to reduce methane levels. Results show improved fan power reduction (23%) and faster computation time for real-time deployment. A double layer BiLSTM model is also used in ref. [62] to optimally reduce CO removal after blasting in a tunneling application. Authors show that BiLSTM is able to properly capture pollutant diffusion behavior and help reduce fan energy consumption by 64.6% over a 1000 s interval.

### 4.3. Existing Limitations, Research Gaps, and Future Promise in the Use of AI for VOD

This section summarizes the results associated with identifying limitations and gaps in the existing literature, as well as specific tasks for which AI can provide additional improvements, thus discussing the answers related to Research Questions Q3 and Q4.

**Overview of AI/ML trends.** This comprehensive review of AI and ML applications in VOD reveals important trends shaping intelligent mine ventilation. Energy efficiency dominates performance metrics, with reported savings ranging from 10.3% to 65.6%. This validates the fundamental VOD premise that demand-responsive ventilation outperforms constant-volume systems. However, the focus on energy may obscure critical dimensions, such as safety outcomes, system reliability, maintenance requirements, and worker acceptance. Hybrid deep learning architectures dominate sensing and forecasting tasks, reflecting increasing recognition of the complex, multi-physics nature of mine ventilation systems. The progression from standalone models (CNN, LSTM, and ANFIS) to sophisticated hybrid configurations (CNN-LSTM, BiLSTM, and Encoder–Decoder LSTM) demonstrates recognition that mine ventilation dynamics require multi-faceted analytical approaches. These models consistently achieve superior performance metrics, with R^2^ values exceeding 0.9. For instance, CNN-LSTM configuration achieved R^2^ of 0.999, with an MAE of 0.065 in airflow prediction, while AMCABN-MEMSA demonstrated an R^2^ of 0.981 for methane concentration forecasting. Most forecasting efforts focus on short-term predictions (2–30 min ahead), aligning with the safety-critical nature of mine ventilation requiring rapid response. However, this reveals a gap in medium-to-long-term predictive capabilities needed for strategic planning and maintenance scheduling. A notable observation is the conspicuous absence of Generative AI techniques. While discriminative models dominate, generative approaches remain unexplored despite capability to address critical limitations, particularly regarding data scarcity and scenario generation for robust testing.

**Sensor placement strategies.** The diversity of proposed methods, including frequency sensitivity analysis, decision rule-based placement, and graphical algorithms, indicates several directions are still being explored in the literature and further studies are needed. In addition, each approach optimizes different priorities: EPAQS maximizes coverage without redundancy, decision trees prioritize operational needs, while independent cut sets focus on mathematical completeness. This heterogeneity indicates that strategy selection must be context-dependent, considering mine geometry, network complexity, budget, and monitoring objectives. There is also a notable absence of work focused on the changes needed in the sensing infrastructure as the mine operation progresses, and whether this can be integrated considering mine planning information.

**Digitalization and data collection challenges.** Several cross-cutting challenges emerge, including limited consideration of data persistence, lack of standardized data collection protocols, and poorly understood temporal resolution requirements. Reported sampling rates vary widely, from seconds to hours, raising questions about the optimal balance between data granularity, computational cost, and predictive accuracy.

**Actuator coordination.** Actuator coordination analysis reveals fundamental trade-offs. Centralized approaches offer global optimization but suffer from single points of failure and communication dependencies, which is problematic in underground environments. Hierarchical control—dividing functions across mine-wide, mining-area, and working-face levels—synthesizes global optimization with resilience and local responsiveness. Future VOD systems will likely adopt multi-layered structures maintaining safety functions during communication disruptions while optimizing system-wide performance. Studies focused explicitly on achieving a robust coordination to face worst-case scenarios are, at the time of this review, still missing.

**Control and decision-making strategies.** The controller landscape reveals evolution from optimization-based methods toward adaptive neuro-fuzzy architectures. Multi-objective optimization approaches, such as MOBWO, report 10.3–21.1% energy reductions through systematic multi-objective search. However, ANFIS-based controllers demonstrate up to 43% laboratory savings and 23% real-world reduction (AMCABN-MEMSA), suggesting a paradigm shift toward adaptive, learning-based control. This reflects neuro-fuzzy advantages: interpretability through rule-based structure, adaptability through continuous learning, and uncertainty handling. Integration with cloud infrastructure further enables real-time updates and remote accessibility, advancing intelligent and responsive VOD systems. At the same time, this trend highlights the need for improved digitalization and reliable communication networks in underground environments. Reports focused on robust control deployment and integrating automatic maintenance and progressive wear in components are also absent from the literature.

**Validation strategies**. A concerning trend is the predominance of simulation-based validation and laboratory-scale experiments. While some models, such as CNN-LSTM, have achieved exceptional field performance (R^2^ = 0.999), most studies rely on computational experiments or CFD-guided platforms. This raises concerns about generalizability when confronted with operational realities, equipment failures, sensor degradation, geological conditions, and human–system interactions. The acknowledgment that signals require outlier filtering emphasizes deployment challenges in noisy environments. On “bad-day” conditions, the literature most often mentions sensor blinding/drift (e.g., post-blast dust); actuator faults (stuck dampers, VFD trips); and noisy/outlier signals, with common responses including short hold-last-trusted windows, alerts to crews, automatic switch to safe fallback flows in affected zones, down-weighting suspect sensors via redundancy checks, and rate caps to avoid overshoot during recovery. However, detailed field validation of these behaviors and consistent reporting metrics remain limited.

**Safety limitations**. To compare beyond energy, studies should report the % time within threshold limit values, the exceedance duration (95th/99th), the override and nuisance-alarm rates, the VOD availability, and the MTBF/MTTR for sensors and actuators. Across the surveyed literature, most works have optimized energy or prediction accuracy, while fail-safe behavior is only briefly noted or left implicit. Where described, fail safe is understood as moving the system to a known safe state when inputs or actions are uncertain, and typically conservative ventilation is achieved using pre-approved higher flows. Only a few papers specify how this transition is managed. A pragmatic synthesis from reported practices is a graded degradation path rather than an on/off switch where normal operation when confidence is high; advisory/confirm modes as confidence dips; constrained control with tighter bounds and slower changes under growing uncertainty; fallback schedules when data are unreliable; and emergency high flow if safety limits are at risk. Human authority remains explicit in these accounts, supported by hard safety limits, time boxing of departures from normal control, and basic audit trails. This trend also highlights the need for Human-CPS designs for VOD implementations.

## 5. Conclusions

Intelligent mine ventilation systems have transitioned from theoretical concepts to deployable technologies. However, realizing full potential requires addressing persistent gaps, such as the standardization of protocols and metrics, extensive real-world validation, human factors integration, robust sensor failure handling, and hybrid architectures maintaining safety during degradations. Although the use of AI-based methods has already reached all the VOD subsystems functionalities, existing work is limited to the use of narrow AI and optimization methods, rather than taking advantage of more recent tools like GenAI and AGI. Future research integrating these tools may allow integrated designs, optimizing further global performance, and exploring what–if scenarios for increased robustness. In addition, many studies considering the use of AI in VOD are still a lower stage of development, considering mostly simulation and laboratory-based studies rather than real-life environment validation. This highlights a need for proving grounds and testbeds available to researchers and industry providers to further advance research and development in this area that can contribute to more transparent evaluation and faster time to market for AI-enabled VOD solutions.

## Figures and Tables

**Table 1:** Research questions.

Q1	For which operational or analytical tasks have AI methods been applied to VOD systems over the past eleven years?
Q2	Which specific AI techniques or models have been employed to support these VOD-related functions or decision-making processes?
Q3	What are the principal limitations and technical challenges associated with current AI-enabled approaches to VOD implementation?
Q4	In which VOD system components or operational domains could AI provide additional improvements in performance, efficiency, adaptability, or system-level intelligence?

**Table 2:** Inclusion and exclusion criteria.

Criteria Type	Included	Excluded
Search Strategy	Queries run in Scopus/Web of Science/IEEE Xplore and major publisher platforms; TITLE-ABS-KEY scope; Boolean + proximity combining (mine ventilation terms) AND (AI terms); NOT filters to remove non-mining contexts (e.g., building/HVAC, medical)	Results from full-text-only searches; records lacking co-occurrence of ventilation and AI terms; hits from non-mining ventilation domains not filtered out
Publication Range	Studies published in 2014 to July 2025 reflecting the evolution of technology in VOD	Studies published before 2014 and not reflective of modern AI or VOD advancements
Record Type	Peer-reviewed journals, industry white papers, textbooks, or peer-reviewed technical conference papers.	Press releases, unpublished theses, blogs, and editorials
Technical Integration	Covers the use of AI across sensing, actuation, control, and decision making for VOD	Focuses only on basic control systems or manual ventilation without intelligent automation or adaptive response
Research Focus	Articles addressing VOD systems, AI-based method in the broad sense of the definition provided in Section 1	Studies that focus solely on surface mining, tunnel construction, or HVAC systems without direct application to underground mines

**Table 3:** Sensor placement strategies.

Inputs	Outputs	Methods	Performance Metrics	Ref.
VNL; NS available; airflow directions; pressure losses; surface roughness	Optimized spatial distribution of fixed air quality sensors; placement for non-regulatory air quality monitors	EPAQS; GA; Modified A * Pathfinding	The pairwise overlap between sensors (average, maximum) and its percentage reduction	[50]
VNL; VNG; sources and sinks; single-junction cut cells; known air volume measurement	NS minimum and optimal location of wind speed sensors; reconstruction of air volume for all tunnels	Independent cut set algorithm; Improved Breadth-First Search (IBFS) over spanning tree; matrixing for algorithm optimization; Gaussian elimination	Error of flow reconstruction; ratio of sensor to tunnel number (RST)	[51]
VNL and network constraints (node air volume balance, air volume wind pressure); NS for wind speed; airflow disturbance; roadway support; distance from inlet outlet; roadway type	Scientific layout of the wind speed sensor; classification conditions for sensor location	DT Algorithm; Entropy Information Theory	Efficiency as associated with information entropy/gain, accuracy, and complexity	[52]
VNG, shortest airflow time; shortest alarm time; cost and location sets	Optimized sensor layout; NS minimum; provides the relationship diagram between the shortest alarm time and extra NS for gas required	Hybrid GA; Discrete Binary Particle Swarm Optimization (GA-DBPSO)	NS minimum required; number of iterations; variance; coverage proportion	[53]
VNG; node types (required placement, alternative points, wind network nodes); shortest airflow time; monitoring effective level; reliability standard	Optimal sensor location schemes; scientific layout	Hybrid Pareto Ant Colony Algorithm (HPACA)	NS minimum required; maximum sensor reliability; proportion of total nodes where sensors are installed	[54]
Ventilation characteristic curves; database at different frequencies; VNL and resistance; time-series data for wind quantity; hazardous gas concentration; gas threshold value; wind resistance	Branch wind quantity/mine ventilator frequency; unique branch resistance model solution; optimal mine ventilator operating frequency	Chebyshev interpolation method (CIM) (for fan curve fitting); Generalized Cross Checking (GCV); Greed Evolution (GE) algorithm	Curve fitting accuracy; safety margin (concentration/threshold); Response time; instability reduction	[28]

Acronyms used: ventilation network layout (VNL), ventilation network graph (VNG), and number of sensors (NS).

**Table 4:** Sensing and forecasting strategies.

Inputs	Outputs	Methods	Performance Metrics	Ref.
Multi-sensor gas readings at a coal face (2 min sampling; correlated neighboring sensors used as spatiotemporal features)	Gas concentration at the target point	Encoder–Decoder LSTM with L1-regularized loss	Mean absolute error (MAE) and accuracy improvement	[55]
Longwall CFD outputs across 6 shearer positions (~31 M cells/location): x, y, z coordinates; airflow velocity (Vx, Vy, Vz); CH_4_ concentration; cell volume; plus distance-to-shearer feature	Real-time 3D explosive methane–air zone prediction (spatiotemporal mapping) with ~2 min latency after ingest	Modified LSTM (3D input adaptation with vector ops); trained/validated on CFD outputs; large-scale spatiotemporal sequence learning	Overall accuracy (87.9–92.4% across shearer–location pairs with training/validation accuracy ~89.1–93.8%); prediction time (2 min)	[56]
Multivariate monitoring: methane sensors, wind speed, temperature, humidity, barometer, machine currents, pipeline pressure/ΔP, cutter speed (V).	Gas concentration 30 min ahead; abnormal emission prediction	Indicators’ dynamic optimization and bi-directional LSTM network used to extract time-series and spatial–topology features; two-layer standard LSTM network	Regression: R^2^ (0.965); MAE (0.039, RMSE achieved 0.046 classification: false alarm rate (FAR) (0.0%), missing alarm rate (MAR) (20.1%), and prediction efficiency (R) (79.9%))	[57]
Gas concentration for CH_4_, CO_2_, CO, O_2_, and H_2_, with 1 h sampling over 153 days	Hour-ahead concentrations of CH_4_, CO_2_, CO, O_2_, and H_2_ in the sealed-off region	t-distributed stochastic neighbor; variational auto-encoder (VAE) for denoising; and bi-LSTM for prediction	Test MSE/MAE: CH_4_: 0.077/0.369; CO_2_: 0.998/1.018; CO: 0.077/0.296; O_2_: 0.298/0.581; H_2_: 0.233/0.549.	[58]
Wind speed and absolute gas inflow	Gas concentration vs. time under different ventilation speeds	DL-Koopman forecasting model	Mean absolute percentage error (MAPE); accuracy; error analysis	[54]
Temperature; humidity; CH_4_; CO_2_; NO_x_; SO_x_	Airflow of the ventilation system	Hybrid 1D-CNN and Long Short-Term Memory (LSTM)	Accuracy; root mean square error (RMSE); MAE; R^2^	[32]
Airflow Q from 21 monitoring points (features evaluated included edges, airflow, wind resistance, total resistance, natural wind pressure)	Airflow at 51 perception points across the mine	Hybrid Convolutional Neural Network (CNN) and Long Short-Term Memory (LSTM)	R^2^, MAE, MAPE, RMSE, RMB	[59]
Airflow at 21 monitoring points from wind-speed sensors; wind speed V combined with airway cross-section S to compute input airflow Q_s_ training data generated via ventilation-network simulations (832 scenarios)	Airflow quantity (m^3^/s) at 51 perception points across the mine (global airflow from partial sensors)	Compared CNN, LSTM, and CNN-LSTM; best is CNN-LSTM multi-output regression	RMSE; MAE; MARE; MAPE	[41]

**Table 5:** Actuator placement strategies.

Method	Operating Principle	Performance Metrics	Ref.
MINLP Model I (optimization with selected fans)	Seeks to minimize ventilation energy use by identifying optimal regulator locations and models and minimizing their number.	Minimizes four objectives: ventilation energy consumption (Z_1_), optimal regulation location (Z_2_), optimal regulation mode (Z_3_), and minimum number of regulators (Z_4_). Achieved energy savings of 65.60% and optimized the regulation network without adding new regulators	[60]
MINLP Model II (optimization without selected fans)	It focuses on determining the required air pressure (H_f_) of fan branches, which is constrained by the requirement that fan pressure must exceed the algebraic sum of air pressures in the same pathway.	Minimizes four objectives: ventilation energy consumption, optimal regulation location, optimal regulation mode, and minimum number of regulators. Directly solvable by conventional solvers.	[61]
Hybrid Spherical Fuzzy-Cloud Model Approach (AHP-based)	Seeks to optimize the selection of booster fans to improve safety and operational efficiency. It employs the analytic hierarchy process (AHP) to structure decision making. Complex spherical fuzzy theory converts expert opinions into superiority scores. Cloud model theory is used for risk analysis and visualization.	Superiority ranking of candidate booster fan solutions (e.g., BF4 > BF3 > BF2 > BF1). Also analyzes risk and optimization potential using cloud model parameters (expectation, entropy, and hyper entropy) for specific indicators.	[61]
Improved Parallel Bare-Bones Particle Swarm Optimization (BBPSO-Para-Improved)	It uses optimization to minimize power consumption and maximize safety in terms of maximal air demand. Airflow positioning is considered within the decision parameters in the optimization problem. An evolutionary algorithm using Gaussian sampling.	Average convergence rate, average calculation time (s), global optimization performance, and convergence efficiency	[27]

**Table 6:** Control and decision-making strategies.

**Inputs**	**Outputs**	Methods	Performance Metrics	Ref.
VNL and its parameters, atmospheric pressure, dry and wet bulb temperature, and wind speed. Constraints on branch airflow/pressure and fan operation constraints also considered	Pareto set of optimized branch airflows and fan pressures minimizing total fan power and pressure-regulation complexity	MOBWO for airflow distribution; graph-theory-based network model; compared to NSGA-II, MOPSO using GD/IGD/spacing/spread	Energy use reduction: generational distance (GD), inverted generational distance (IGD), GD, spacing, and spread. Case study yields 10.3–21.1% FP reduction	[19]
Air pressure (P), air velocity (v), air resistance and air quantity, flow rate (Q), CH4 concentration (M), fan power (FP), and dilution time t	(1) optimal FP for dilution; (2) predicted dilution time of methane concentration to allowable levels. System provides combinations of FP and decision-making time	Two Sugeno-type ANFIS models to predict optimal fan power and dilution time; Model-1 inputs: P, Q, M with 45 fuzzy rules. Model-2 inputs: P, Q, M, FP with 135 rules	Test accuracy: R^2^ = 0.84 (optimal FP), R^2^ = 0.96 (dilution time). Laboratory VOD energy savings: up to 43% vs. conventional worst-case sizing; case-by-case savings depend on L/D and CH_4_ levels	[26]
Time-series from CFD/field for the heading area: CO concentration, air volume/velocity, temperature, and relative humidity; past window: 30 s; future horizon: 10 s	Next-step CO concentration (10 s ahead), which is then converted to required air volume for DCV control	Demand-controlled ventilation (DCV) based on DL. BiLSTM for prediction with adaptive moment estimation (Adam) optimizer; SHapley Additive exPlanation (SHAP) method is used as explainable AI	MSE and R^2^ for training/testing; CO removal efficiency (RE), fan energy consumption (W); coefficient of ventilation performance (COVP); maximum average CO concentration; ventilation time	[62]
Real-time sensor data for CH_4_ concentration, fan speed, and power calculation	Predicted optimal fan speed (P′), which is used to estimate fan power and provide commands to adjust the variable speed drive (VSD) in real time	Hybrid DL model combining advanced multi-head cross attention-based BiLSTM (AMCABN) and MEMSA for prediction and optimization, respectively	Energy consumption/power reduction (kW and %); convergence rate/speed; accuracy and precision, MSE, RMSE, and R^2^; CH_4_ reduction time (18 min); FP reduction (23%); maximum fan power reduction (350 kW)	[63]
Air quality, air pressure, air power, efficiency, productivity, safety, flexibility, noise, vibration, and operating cost	Ranked fan solutions for decision-making and risk analysis associated with optimization potential	Hybrid spherical fuzzy-cloud model combination: improved AHP for weighting and structuring decisions, complex spherical fuzzy theory for ranking alternatives, and cloud model theory for risk assessment and optimization	Consistency ratio (CR), superiority scores, cloud model parameters (entropy, hyper entropy and expectation); external validation: compared to the fuzzy TOPSIS (FT) method, showing high consistency in ranking	[61]
